# Knee Ligament Injuries in the National Football League: Impact of Field Surface Type During the 2020-2023 Seasons

**DOI:** 10.7759/cureus.76425

**Published:** 2024-12-26

**Authors:** William F McCormick, Mitchell J Lomis, Christian B Cooper, Matthew Yeager, Nicholas J Tsavaris, David Patch

**Affiliations:** 1 Orthopedic Surgery, University of Georgia, Athens, USA; 2 Orthopedic Surgery, AU/UGA Medical Partnership, Augusta, USA; 3 Orthopedic Surgery, AU/UGA Medical Partnership, Athens, USA; 4 Orthopedic Surgery, University of South Alabama College of Medicine, Mobile, USA; 5 Orthopedic Surgery, University of Alabama at Birmingham School of Medicine, Birmingham, USA

**Keywords:** acl, artificial turf, football, ligament, surface

## Abstract

Introduction

A subject of ongoing debate within the National Football League (NFL) community revolves around the comparative risk of anterior cruciate ligament (ACL) injuries on natural versus artificial turf field surfaces. There have been mixed results as to whether there is a difference in injury rates depending on the playing surface and what factors might play a role in affecting these rates.

Methods

This study aims to compare the incidence of in-game knee ligament tears in the NFL during the 2020-2023 seasons. Data was collected from publicly available resources to determine and classify specific injury characteristics such as ligament, injury date, location, and field surface type. Only in-game knee ligament tears, including those occurring in pre-season games, were included. Significance was determined using two-sample T-tests.

Results

Over the 2020, 2021, 2022, and 2023 seasons, there were 149 ligament tears, with 58 occurring on natural turf (38.9%) and 91 occurring on artificial turf (61.1%). Over the course of the same four years, 1213 games were played, with 524 (43.2%) games on natural turf and 689 games on artificial turf (56.8%). Accounting for the number of games played on each surface, natural turf saw a rate of 0.111 knee ligament tears per game, while artificial turf saw 0.132 ligament tears per game.

Conclusion

Over the course of the study period, we determined that there was no significant difference between the rates of in-game knee ligament tears on natural and artificial turf playing surfaces. Our study shows that regardless of differences in composition and other playing surface features, there is ultimately no difference in the incidence of knee ligament tears. Playing surfaces should still be monitored and regulated, and the incidence of other injuries, such as concussions, as well as player preference, should be considered when considering the ideal surface.

## Introduction

A subject of much ongoing debate within the National Football League (NFL) community revolves around the comparative risk of anterior cruciate ligament (ACL) injuries on artificial turf versus natural grass field surfaces. Recent data from the NFL Players Association has brought this issue to the forefront, indicating a significantly higher incidence of ACL injuries on turf fields compared to grass fields [1-2]. In response to these findings, the NFL has expressed a nuanced perspective, suggesting that the relationship between playing surface type and ACL injury rates is multifaceted. Statements from the league's committee and experts from the NFL Players Association have emphasized that the solution to this complex challenge may not be as straightforward as solely advocating for natural grass fields [3]. Indeed, certain previous studies have indicated artificial turf surfaces showing lower ACL injury rates than grass fields, complicating the issue further [4].

Echoing this sentiment, influential figures within the NFL, such as Dallas Cowboys owner Jerry Jones, have minimized concerns regarding playing surface type, citing league statistics that purportedly do not indicate significant issues with artificial turf surfaces compared to natural grass in terms of ACL injury rates [5]. However, the bulk of recent research studies on the topic have provided compelling evidence regarding the association between playing surface type and ACL injury rates. For instance, a study conducted by Mack et al. concluded that NFL play on synthetic turf resulted in a higher incidence of ACL injuries per play compared to natural grass [2]. This study proposed that synthetic turf surfaces do not release cleats as readily as natural turf, and the concomitant increased loading on the foot contributes to the incidence of lower body injuries. Similarly, research in National Collegiate Athletic Association (NCAA) football has revealed a significant association between artificial turf surfaces and ACL injuries, with lower NCAA divisions showing higher rates of ACL injuries during competitions on artificial turf versus natural grass [6]. The abundance of literature-backed associations between artificial turf surfaces and ACL tears underscores the necessity for comprehensive investigations into the incidence and severity of ACL injuries relative to playing surface type within the NFL context [7]. This is crucial in order to mitigate any potential elevated risk of significant injuries for athletes playing on these surfaces. Despite the wealth of research on injury frequencies and specific injury types, there remains a gap in the literature regarding the incidence of severe or season-ending ACL injuries in relation to field playing surfaces within the NFL context. The purpose of this study is to fill this gap by examining the relationship between season-ending ACL injuries on artificial turf and natural grass fields among NFL players from 2020 to 2024. 

## Materials and methods

Injury information on NFL players was obtained using publicly available data (fantasydata.com [8], profootballreference.com [9], spotrac.com [10], ESPN.com [11], NFL.com [12]) from the 2020, 2021, 2022, and 2023 seasons. Characteristics of each injury were researched and recorded, as well as the specific game, date, stadium, and playing surface where the injury occurred. Only injuries involving one or more knee ligaments (anterior cruciate, posterior cruciate (PCL), medial collateral (MCL), lateral collateral(LCL)) were included. Data was entered into Excel and analyzed using two-sample T-tests with a significance threshold of p=0.05. Note that any injury resulting in multiple torn ligaments was counted as a single injury, resulting in a greater number of “tears” than “injuries.” This is reflected throughout this manuscript through the deliberate use of the word “injury” rather than “tear.”

Inclusion-exclusion criteria

Only in-game injuries, including those occuring in pre-season games, were included. However, post-season/playoff injuries were excluded. Sprains, strains, and injuries to other anatomical features of the knee, such as tendons and menisci, were excluded unless concomitantly involving a knee ligament tear. Any injury that could not be clearly identified was excluded.

Field surface classification

A surface with any proportion of artificial turf was included in the artificial turf category. This includes surfaces comprised of mixtures of artificial and natural turf. Only fields with 100% natural grass were included in the natural grass category.

## Results

**Figure 1:**
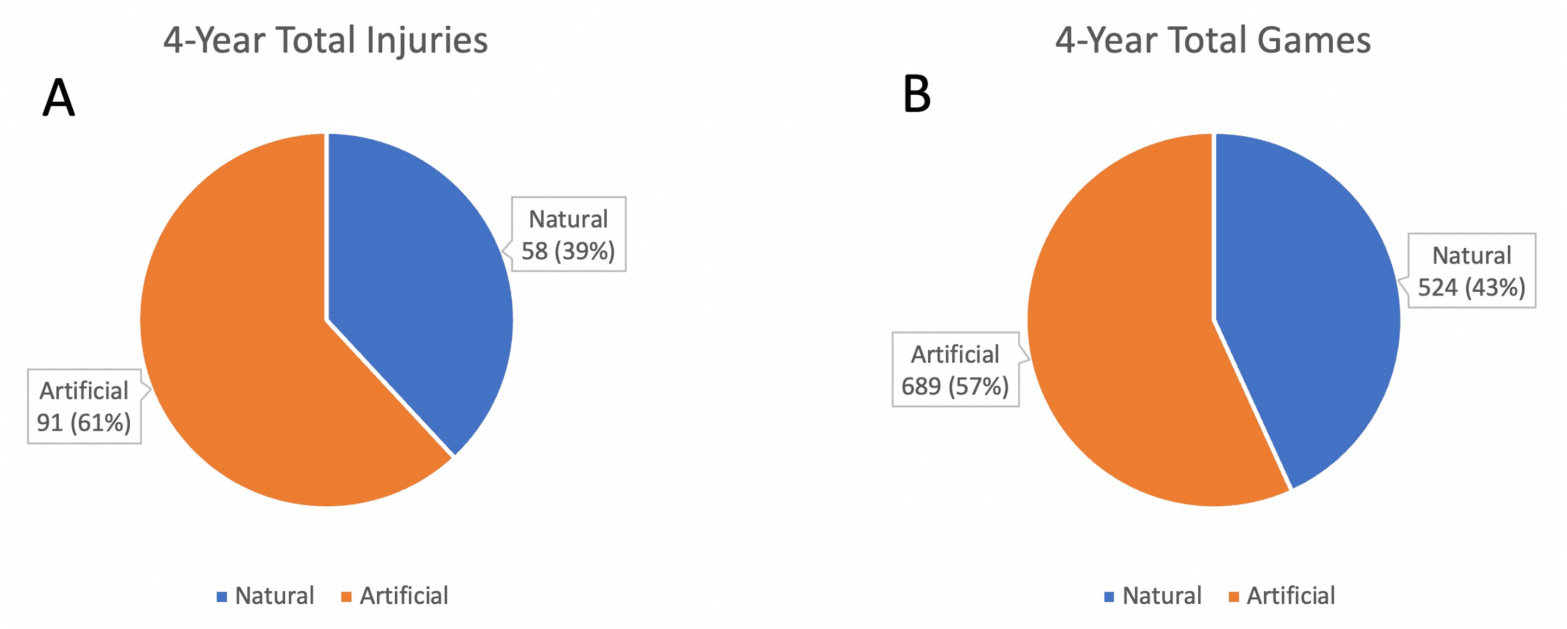
Relationship of each surface in comparison to total number of injuries. A: (n=149) and total number of injuries; B: (n=1213) and total number of games over the four-year study period.

Over the course of the 2020, 2021, 2022, and 2023 seasons, there were a total of 149 knee ligament tear injuries. Of those injuries, 58 occurred on natural surfaces, while 91 occurred on artificial turf surfaces. In those four years, 524 games were played on natural turf, and 689 games were played on artificial turf, as shown in Figure 1. Over this four-year period, there was a rate of 0.111 injuries per game on natural turf and 0.132 injuries per game on artificial turf.

 In 2020, natural turf accounted for 13 ligament injuries over 120 games (0.108/game), while 20 in 136 games (0.147/game) occurred on artificial turf. In 2021, natural turf saw 19 injuries in 140 games (0.136/game), while artificial turf saw 27 in 182 games (0.148/game). In 2022, 11 injuries occurred in 136 games on natural turf (0.081/game), and 27 injuries occurred in 178 games on artificial turf (0.152/game). Lastly, in 2023, natural turf saw 13 knee ligament injuries in 128 games (0.102/game), and artificial turf saw 17 injuries in 193 games (0.088/game). Overall, from 2020-2023, 58 knee ligament injuries occurred in 524 games on natural turf (0.111/game), and 91 ligament injuries occurred in 689 games on artificial turf (0.132/game). Individual ligament tear types and frequency are shown below in Table 1.

**Table 1:** Individual ligament tears and the surface on which they were torn. Note that the total number of ligament tears here is greater than the total of injuries reported for each surface due to the occurrence of multi-ligament injuries.

Year	Ligament	Natural	Artificial	Total
2020	Anterior cruciate ligament	13	17	30
	Medial collateral ligament	1	4	5
	Posterior cruciate ligament	0	3	3
	Total	14	24	38
2021	Anterior cruciate ligament	16	26	42
	Medial collateral ligament	4	2	6
	Posterior cruciate ligament	1	0	1
	Total	21	28	49
2022	Anterior cruciate ligament	10	22	32
	Medial collateral ligament	1	7	8
	Posterior cruciate ligament	0	0	0
	Total	11	29	40
2023	Anterior cruciate ligament	11	16	27
	Medial collateral ligament	2	1	3
	Posterior cruciate ligament	0	0	0
	Total	13	17	30

Two-tailed T-tests comparing the rates of ligament tears per game on natural turf versus artificial turf for each individual year and for the combined four-year period resulted in p-values of 0.382 in 2020, 0.854 in 2021, 0.116 in 2022, 0.709 in 2023, and 0.379 for the combined four-year period respectively.

## Discussion

Over the course of the 2020, 2021, 2022, and 2023 seasons, a total of 149 knee ligament tear injuries were recorded. Our analysis indicates a higher incidence of knee ligament injuries on artificial turf surfaces, with 91 injuries occurring on artificial turf compared to 58 on natural grass. When considering the number of games played on each surface, the injury rate per game was 0.132 for artificial turf and 0.111 for natural grass. Overall, there was a greater number of injuries of all types occurring on artificial turf. 

One explanation for this difference is the recent increase in fields using artificial turf. As of the end of the 2023 season, there were 19 teams playing on artificial turf and only 13 playing on natural grass, roughly 59.4% and 40.6%, respectively. This closely reflects the percentage of injuries occurring on each surface in relation to total injuries, with 56.8% of injuries occurring on artificial turf and 43.2% on natural grass, a relationship demonstrated below in Figure 1. Although the difference in injury rates between the two surfaces did not prove statistically significant (combined p-value of 0.379), these findings still underscore the importance of the NFL continuing to monitor and evaluate the safety of its playing surfaces. 

While the statistical insignificance of the difference in injury rates suggests that the relationship between playing surface and injury risk is complex, other factors allowing classification of surfaces beyond just natural and artificial should be considered in the conversation of player safety. Understanding the specific factors that may contribute to higher injury rates on artificial turf, such as the type of turf, field-maintenance practices, as well as player equipment will prove crucial in developing strategies to enhance player safety.

It should be noted that the current body of evidence offers little information on the topic of discussion, mostly focusing on other levels of competition or generalizing methodology to focus on lower extremity injuries (LEI) as a whole [2,4,6,13]. Additionally, one study looked at ligament sprains, rather than tears, in the knee [14]. 

Hershman et al. found that the ACL sprain rate was higher on artificial turf but not the rate of MCL sprains [14]. This is an interesting point of contrast from our study that focused on knee ligament tears as one entity rather than specific types of sprains in the knee. Hershman et al. and one other study used injury data from earlier NFL seasons (2000-2009 and 2012-2016, respectively), which both occurred prior to the domination of the artificial turf field in the NFL [2,14]. There has been a substantial increase in games played on artificial turf, which can contribute to a difference in results. Additionally, the composition and quality of turf fields have changed significantly since it was first used. Previous studies have denoted that turf qualities, such as fiber density and surface compaction, affect extremity joint loading. Differences in joint loading have been continuously noted as the theory behind differences in injury rates between various field surfaces [2,15].

The design of studies that have investigated generalized lower extremity injury differs substantially from this one, however it should be noted that one study took data from 2020-2021, overlapping with our study period. In this study, Venishetty et al. found that LEI rates per game were higher on artificial turf and additionally found that when controlled for season-ending injuries, there was a significant increase in injury prevalence on artificial turf compared to natural grass [13]. However, as we have seen in our study, a narrow scope of purely season-ending injuries leads to a small sample size, which diminishes power. It should be noted that the results of previous studies are mixed regarding the topic of our study. Comparing our results to these studies is difficult, however each is important to the comprehensive understanding of this topic of this topic.

This study has several limitations that should be acknowledged. The reliance on publicly available data sources may have introduced inconsistencies or inaccuracies in injury reporting. Furthermore, the study did not account for other factors that could influence injury rates; these include things such as individual player positions, weather conditions at the time of injuries, or variations in the types of artificial turf used by stadiums. Additionally, the relatively small sample size and the focus solely on knee ligament injuries may limit the generalizability of our findings.

Future research should address these limitations by incorporating more comprehensive and detailed data collection methods. Further studies could explore the biomechanical differences between playing surfaces in greater depth and examine the effectiveness of various injury prevention strategies. Expanding the scope to include other types of injuries and considering factors such as player position and specific turf characteristics could provide a more holistic understanding of the relationship between playing surface and injury risk in the NFL.

## Conclusions

Our results show that, while knee ligament tear injuries are more common on artificial turf, the difference is not statistically significant when accounting for the increased amount of play seen on this surface. Variation occurs between all surfaces, even of the same type, artificial or natural. Surfaces should continue to be regulated and monitored for traits such as hardness, and players' preferences should be considered for qualities that are not quantifiable. 
